# Breast cancer risk in ataxia telangiectasia (AT) heterozygotes: haplotype study in French AT families

**DOI:** 10.1038/sj.bjc.6690460

**Published:** 1999-06

**Authors:** N Janin, N Andrieu, K Ossian, A Laugé, M-F Croquette, C Griscelli, M Debré, B Bressac-de-Paillerets, A Aurias, D Stoppa-Lyonnet

**Affiliations:** Institut Gustave Roussy, 39 rue Camille Desmoulins, 94805 Villejuif Cedex, France; U351 Inserm, Institut Gustave Roussy, 39 rue Camille Desmoulins, 94805 Villejuif Cedex, France; Institut Curie, Unité de Génétique Oncologique, Laboratoire de pathologie moléculaire des cancers, 26 rue d'Ulm 75231 Paris Cedex 5, France; Centre Hospitalier Feron-Vrau, 219 & 329 Boulevard Victor Hugo, BP 255, 59019 Lille, Cedex, France; Hôpital des Enfants Malades, Service d'Immuno-Hematologie pédiatrique, 149 rue de Sèvres, 75743 Paris, Cedex 15, France

**Keywords:** ataxia telangiectasia heterozygosis, breast cancer risk, family study

## Abstract

Epidemiological studies in ataxia telangiectasia (AT) families have suggested that AT heterozygotes could have an increased cancer risk, especially breast cancer (BC) in women. It has also been suggested that an increased sensibility of AT heterozygotes to the effect of ionizing radiation could be responsible for the increased BC risk. BC relative risk (RR) estimation in AT heterozygotes within families ascertained through AT children is presented here. Family data collected included demographic characteristics, occurrence of cancers, past radiation exposures and blood samples. DNA samples were studied using seven ATM linked microsatellites markers allowing AT haplotypes reconstitution. The relative risk of BC was assessed using French estimated incidence rates. A significant increase risk of BC is found among obligate ATM heterozygotes with a point estimate of 3.32 (*P* = 0.002). BC relative risk calculated according to age is significantly increased among the obligate ATM heterozygotes female relatives with an age ≤ 44 years (RR = 4.55, *P* = 0.005). The BC relative risk is statistically borderline among the obligate ATM heterozygote female relatives with an age ≥ 45 years (RR = 2.48, *P* = 0.08). The estimated BC relative risk among ATM heterozygotes is consistent with previously published data. However, the increased risk is only a little higher than classical reproductive risk factors and similar to the risk associated with a first-degree relative affected by BC. © 1999 Cancer Research Campaign


*British Journal of Cancer* (1999) **80**(7), 1042–1045
© 1999 Cancer Research Campaign
Article no. bjoc.1999.0460

### Breast cancer risk in ataxia telangiectasia (AT) heterozygotes: haplotype study in French AT families

**N Janin[1]*, N Andrieu[2]*, K Ossian[1], A Laugé[3], M-F Croquette[4], C Griscelli[5], M Debré[5], B Bressac-de-Paillerets[1], A Aurias[3] and D Stoppa-Lyonnet[3]**

[1]Institut Gustave Roussy, 39 rue Camille Desmoulins, 94805 Villejuif Cedex, France; [2]U351 Inserm, Institut Gustave Roussy, 39 rue Camille Desmoulins, 94805 Villejuif Cedex, France; [3]Institut Curie, Unité de Génétique Oncologique, Laboratoire de pathologie moléculaire des cancers, 26 rue d'Ulm 75231 Paris Cedex 5, France; [4]Centre Hospitalier Feron-Vrau, 219 & 329 Boulevard Victor Hugo, BP 255, 59019 Lille, Cedex, France; [5]Hôpital des Enfants Malades, Service d'Immuno-Hematologie pédiatrique, 149 rue de Sèvres, 75743 Paris, Cedex 15, France

**Summary** Epidemiological studies in ataxia telangiectasia (AT) families have suggested that AT heterozygotes could have an increased cancer risk, especially breast cancer (BC) in women. It has also been suggested that an increased sensibility of AT heterozygotes to the effect of ionizing radiation could be responsible for the increased BC risk. BC relative risk (RR) estimation in AT heterozygotes within families ascertained through AT children is presented here. Family data collected included demographic characteristics, occurrence of cancers, past radiation exposures and blood samples. DNA samples were studied using seven ATM linked microsatellites markers allowing AT haplotypes reconstitution. The relative risk of BC was assessed using French estimated incidence rates. A significant increase risk of BC is found among obligate ATM heterozygotes with a point estimate of 3.32 ($P$ = 0.002). BC relative risk calculated according to age is significantly increased among the obligate ATM heterozygotes female relatives with an age ≤ 44 years (RR = 4.55, $P$ = 0.005). The BC relative risk is statistically borderline among the obligate ATM heterozygote female relatives with an age ≥ 45 years (RR = 2.48, $P$ = 0.08). The estimated BC relative risk among ATM heterozygotes is consistent with previously published data. However, the increased risk is only a little higher than classical reproductive risk factors and similar to the risk associated with a first-degree relative affected by BC.

**Keywords:** ataxia telangiectasia heterozygosis; breast cancer risk; family study

Epidemiological studies of ataxia telangiectasia (AT) families have suggested that AT heterozygotes could have an increased cancer risk, especially for breast cancer (BC) in women (Swift et al, 1987, 1991; Pippard et al, 1988; Børresen et al, 1990; Morrell et al, 1990; Athma et al, 1996; Stankovic et al, 1998). The estimation of this increased BC risk assessed from the combined analysis of available data in 1994 was 3.9 (Easton, 1994). In Europe, two out of three studies (Pippard et al, 1998; Børresen et al, 1990; Stankovic et al, 1998) have shown a significant increased risk of BC but with wide confidence intervals (Stankovic et al, 1988; Børresen et al, 1990). Moreover, it has been suggested that an interaction between AT heterozygosis and ionizing radiation exposures could be involved in the increase in BC risk (Swift et al, 1991). However, no data have been published that sustain this hypothesis. The gene for AT (ATM) was identified in 1995 (Savitsky et al, 1995), allowing the identification of ATM heterozygotes in families with an AT-affected child, through segregation of AT-linked haplotypes. Thus an epidemiological study of cancer risks associated with AT heterozygosis collecting information on ionizing radiation exposures has been performed in France. In the present paper, BC relative risk estimation in AT heterozygotes within AT children families is presented.

*Received 4 January 1999*
*Revised 4 January 1999*
*Accepted 6 January 1999*

*Correspondence to:* N Andrieu

### DATA COLLECTION AND METHODS

A family study of the AT children population was carried out in France from June 1994 to February 1997. AT children were recruited by paediatricians who have been surveying this population since early childhood and cytogeneticists who have contributed to their disease diagnosis. AT children were eligible if their family was living in France at the time of the study. For each participant who signed a consent form, a blood sample was taken; and a questionnaire was administrated by a physician to all adult relatives. A blood or a buccal cell sample was taken from the AT children and their siblings with parental agreement.

Demographic characteristics (gender, date of birth and, if deceased, age at death and cause of death) and the occurrence of BC and any other cancer, including age at diagnosis and places of medical care were collected from first-degree (parents and siblings of AT child), second-degree (uncles, aunts and grandparents) and third-degree (granduncles and -aunts, and great-grandparents, cousins of AT child) relatives. Epidemiological data on first- and second-degree relatives aged 18 years or over concerned medical history, exposure to medical and professional radiation and detailed reproductive factors for females.

All contacted families, with the exception of one, consented to participate. Thirty-four French families were recruited. AT children were aged from 3 to 32 years. Eighteen of 29 breast malignancies reported in families could be confirmed by pathological

*Authors contributed equally to this work.

**Table 1** ATMheterozygosis (ATM het) status repartition among female relatives of the 34 families

| | A priori probability approach | Mixed approach | | |
|---|---|---|---|---|
| **ATM het status** | **No. of females (%)** | **No. of females (%)** | **Mean age (s.d.)** | **Person-years** |
| Obligate | 40 (5.6) | 115 (16.2) | 44.2 (19.5) | 5079 |
| 50% | 195 (27.3) | 201 (28.3) | 45.2 (26.6) | 9085 |
| 25% | 344 (48.2) | 107 (15.1) | 48.5 (23.5) | 5192 |
| 12.5% | 117 (16.4) | – | | |
| Obligate non | 18 (2.5) | 288 (40.5) | 46.8 (25.4) | 13468 |
| All females | 711 | 711 | 46.2 (24.6) | 32823 |

s.d. = standard deviation.

**Table 2** BC risk according to ATM het status among female relatives of the 34 families

| | Mixed approach | | | |
|---|---|---|---|---|
| **ATM het status** | **O** | **E** | **O/E** | **95% CI** |
| Obligate | 9 | 2.71 | 3.32 | (1.75–6.38) |
| 50% | 5 | 6.24 | 0.80 | (0.33–1.92) |
| 25% | 3 | 3.42 | 0.88 | (0.28–2.73) |
| Obligate non | 11 | 9.26 | 1.19 | (0.66–2.15) |

O = observed number of BC cases; E = expected number of BC cases.

records and, in all of those, there was complete agreement between the case report and the pathological record.

Genotyping at the AT locus has been performed studying seven polymorphic microsatellite markers, six of which are flanking – two centromeric (D11S1817, D11S1819), four telomeric (D11S1778, D11S1294, D11S2180, D11S2178) and one intragenic (D11S2179). These loci have been selected in view of the maps previously reported (Savitsky et al, 1995; Laake et al, 1997), encompassing a region of approximately 2 megabases. Haplotypes were constructed from the observed segregation of the multiple, closely linked markers in the extended families assuming a minimum number of genetic recombinants. The attribution of the AT heterozygote status was based on the presence of one of the haplotypes of the affected child, or children, of the family. Given the small genetic distance between the markers and the ATM gene and the elevated frequency of heterozygozity (66%) at the studied loci in our sample, the risk of misleading attribution of the ATM heterozygosis (ATM het) status was toward the null.

Where ATM het status was not determined by molecular approach, the a priori probability that the individual with the undetermined status shares AT mutation with their closest ATM obligate heterozygote relative was calculated. This mixed approach provided four classes of AT children relatives: ATM obligate, 0.5 ATM and 0.25 ATM heterozygotes, and ATM obligate non-heterozygotes. The a priori probability of AT children siblings for whom there were no DNA sample equals 0.66 and concerns a small number of AT children relatives. Thus, in order to limit the number of classes they were included in the 0.5 ATM heterozygotes category.

Relatives of AT children were considered at risk from the date of their conception since ATM het status is defined from this moment. However, as the French incidences of cancers were available from age 25 years, the relatives of AT children were considered at risk from 25 years of age to either age at interview or age at death for the non-affected-by-BC subjects, or age at diagnosis for the affected-by-BC subjects. When age at diagnosis of BC was unknown, age at death was used. The relative risk (RR) of BC association with ATM het status was assessed by the ratio of the observed number of BC cases (O) and the expected number of BC cases (E) in the AT families (Breslow and Day, 1987). The calculation of the expected number of BC cases was performed from the French estimated incidences of BC between 1978 and 1987 (Benhamou et al, 1990; De Vathaire et al, 1996) per 5-year age band using the Fortran program PYRS (Coleman et al, 1986). The RR 95% confidence interval (CI) was calculated as proposed by Breslow and Day (1987).

### RESULTS

The 34 families included 1429 persons with a mean number of 42 persons per family (s.d. = 19). DNA samples from 401 individuals were studied. In addition, classification of an extra 412 individuals as ATM obligate heterozygotes or ATM obligate non-heterozygotes was made possible by the mixed approach. The AT children siblings for whom there was no DNA sample were very few (12

**Table 3** BC risk according to ATM het status and age among female relatives of the 34 families

| | Age of female relatives ≤ 44 years | | | | Age of female relatives ≥ 45 years | | | |
|---|---|---|---|---|---|---|---|---|
| **ATM Het status** | **O** | **E** | **O/E** | **95% CI** | **O** | **E** | **O/E** | **95% CI** |
| Obligate | 5 | 1.10 | 4.55 | (1.89–10.9) | 4 | 4.61 | 2.48 | (0.93–6.61) |
| 50% | 0 | 1.64 | 0.00 | – | 5 | 4.60 | 1.09 | (0.45–2.62) |
| 25% | 2 | 0.99 | 2.02 | (0.51–8.08) | 1 | 2.44 | 0.41 | (0.06–2.91) |
| Obligate non | 2 | 2.60 | 0.77 | (0.19–3.08) | 9 | 6.66 | 1.35 | (0.70–2.59) |

O = observed number of BC cases; E = expected number of BC cases.

*© 1999 Cancer Research Campaign*

out of 44 with a mean age of 17 years), and they were included in the 0.5 ATM heterozygotes category. Among the 1429 persons, 29 BC cases had been diagnosed between 1943 and 1996. All cases with the exception of one were female, that is 28 female cases. Ten BC cases were ATM heterozygotes, 11 were not ATM heterozygotes. For the remaining eight BC cases, the ATM status was uncertain with an a priori probability of 0.5 for five and 0.25 for three. Among the 34 families, at least one BC occurred in 20 families. In 14 families only one BC case occurred, in four families two BC cases occurred, in one family three cases, and in another family four cases of BC occurred. The male case was ATM heterozygote with a BC diagnosed in 1972 at 55 years of age.

The age-range of female BC cases at diagnosis was 35–97 years. Among obligate ATM heterozygote female BC cases, diagnoses occurred between 1969 and 1995. Among the others, ATM het status female BC cases, 16 were diagnosed between 1943 and 1995, three had an unknown date of diagnosis, and thus year of their death was used. The three BC cases with an unknown date of diagnosis died at 35, 55 and 70 years respectively, their respective ATM het status is 0.25, 0 and 0.5.

Female relatives of the 34 families are described in Table 1 according to ATM het status comparing the a priori probability approach to the mixed approach (i.e. haplotype study or a priori probability for those whose het status was undetermined by molecular approach). The obvious interest of the mixed approach is an increase by three of the obligate ATM heterozygote number, but also an increase by 16 of the obligate non-ATM heterozygote number. The mean age of the women across ATM het status is similar.

In Table 2, the BC relative risks according to ATM het status among the female relatives are shown. A significant increased risk of BC is found among obligate ATM heterozygotes with a point estimate of 3.32 and a 95% CI of 1.75–6.38 ($P$ = 0.002). A slight non-significantly increased BC risk is found among obligate non-ATM heterozygotes (O/E = 1.19, $IC_{95\%}$ = 0.66–2.15). The BC risk among other ATM het status groups are not significant with similar point estimates (0.5 class: O/E = 0.80, $IC_{95\%}$ = 0.33–1.92; 0.25 class: O/E = 0.88, $IC_{95\%}$ = 0.28–2.73).

In Table 3, the BC relative risks are calculated according to ATM het status and age of the female relatives. Among female relatives with an age equal to or less than 44 years, a significant increase risk of BC is found among obligate ATM heterozygotes with a point estimate of 4.55 and a 95% CI of 1.89–10.9 ($P$ = 0.005). A non-significant decrease BC risk is found among obligate non-ATM heterozygotes (O/E = 0.77, $IC_{95\%}$ = 0.19–3.08). Among female relatives with an age equal to or more than 45 years, a statistically borderline increase risk of BC is found among obligate ATM heterozygotes with a point estimate of 2.48 and a 95% CI of 0.93–6.61 ($P$ = 0.08). A non-significant increase BC risk is found among obligate non-ATM heterozygotes (O/E = 1.35, $IC_{95\%}$ = 0.70–2.59). The BC risk among the other ATM het status groups remains non-significant when taking age into account with variable point estimates and wide confidence intervals (0.5 class: among females ≤ 44 years, zero observed BC case, among females ≥ 45 years O/E = 1.09, $IC_{95\%}$ = 0.45–2.62; 0.25 class: among females ≤ 44 years, O/E = 2.02, $IC_{95\%}$ = 0.51–8.08; among females ≥ 45 years O/E = 0.41, $IC_{95\%}$ = 0.06–2.91).

### DISCUSSION

The finding of this study is in agreement with the increased BC risk previously detected among ATM heterozygotes (Swift et al, 1987, 1991; Pippard et al, 1988; Børresen et al, 1990; Morrell et al, 1990; Athma et al, 1996). The estimated BC relative risk of 3.32 is consistent with Athma et al's (1996) estimate (RR = 3.8) and Easton's (1994) combined estimate (RR = 3.9). The point estimate of BC risk appears higher among female relatives less than 44 years of age than among those more than 45 years of age. This result cannot be affected by a possible misclassification of the three BC cases with an unknown age at diagnosis, none of them being ATM heterozygotes. This result is the opposite to that of Athma et al's (1996) and FitzGerald et al's findings (1997). Indeed, FitzGerald et al (1997) did not find evidence for an increase frequency of ATM heterozygotes among women with early onset of BC. However, let us note that given the low estimated frequency of ATM carriers, they had little chance of detecting an increase risk (40% chance for a fourfold increase in risk and 6% for a twofold increase) (Bebb et al, 1997; Bishop and Hopper, 1997). In the same way, Athma et al (1996) found an increased risk of BC for ATM heterozygotes and even higher at older ages (60 or older).

A possible bias in family recruitment due to a higher participation of families with a BC may be suggested. Indeed, the slight non-significant increase of BC risk among obligate non-ATM heterozygotes may reflect a possible ascertainment bias. However, the BC higher risk among the obligate ATM heterozygotes remains, even when adjusted (i.e. adjusted RR ≈ 2.8).

The slight non-significantly decreased BC risk found among the groups at 50% or 25% risk to be ATM carriers could reflect a bias towards a higher proportion of known heterozygosis status for BC cases than for the non-BC cases leading to an overestimation of obligate ATM carriers BC risk. Indeed, 29% (eight out of 28) of BC cases have an uncertain ATM carrier status against 44% of non-BC cases (300 out of 683). However, the observed decreased BC risk is not statistically significant and the obligate ATM carriers BC risk may not be dramatically overestimated.

The declared BCs among families have been verified for more than the majority. Moreover, BC has been found to be reported with great accuracy in numerous studies. In particular, Theis et al (1994) found concordance of 99% between case report and the pathological report. However, the non-affected-by-cancer individuals in families have not been checked because of lack of a national French registry, although it has been estimated that 98% of negative families' history were correct (Aitken et al, 1995). A poor sensitivity of self-reported family history of BC (i.e. under reporting) may lead to an under-estimate of the BC relative risk across heterozygosis status classes.

The calculations of expected numbers of BC are known to be sensitive to the reference population used. Because national incidence data are not available in France, estimated incidences of BC between 1978 and 1987 were used as the reference population in this study (Benhamou et al, 1990; De Vathaire et al, 1996). This might induce an over-estimate of the expected numbers of BC among exposed women before 1978 (mostly grandmothers, grand-aunts and great-grandmothers of AT children) and an under-estimate of this number among exposed women after 1987 (mostly mothers and aunts) since the BC incidence rate has been increasing in West European countries for a number of decades (Parkin et al, 1993). This may not explain the increased risk of BC among all of obligate AT heterozygotes but might partially explain the observed difference in BC relative risk between younger and older female relatives. For this reason, comparison between the BC relative risks of young and old ATM obligate heterozygote females is more

*© 1999 Cancer Research Campaign*

suitable, adjusted on respective obligate non-ATM heterozygotes BC relative risks. Thus, an observed difference in the point estimates remains with a BC risk of 5.9 among the younger ATM obligate heterozygote female relatives and 1.8 among the olders. Since neither shows convincing difference in RR estimated by age bands given the RR confidence intervals, other studies are needed to clarify this point.

Although still imprecise, the detected increased risk may have a point estimate of about 3, which is only a little higher than classical reproductive risk factors (i.e. a young age at menarche, a nulliparity or a late age at first childbirth etc) and similar to the risk associated with a family history of BC among first-degree relatives (Kelsey and Horm-Ross, 1993). However, the previous suggestion that the ATM heterozygotes would be highly ionizing-radiation sensitive (Swift et al, 1987) is still unconfirmed. Indeed, studies on highly clinical-radiosensitive BC patients did not show evidence for an elevated ATM heterozygote rate (Appleby et al, 1997; Shayeghi et al, 1998). Thus it does not appear necessary, so far, to subject women with ATM het status to any different screening program which is not already available to women with a first-degree relative affected by BC. Nevertheless, further studies are needed for assessing the BC risk among ATM heterozygotes according to their past ionizing-radiation exposures. Indeed, such studies may allow us to improve the understanding of the underlying mechanisms involved in the observed increased BC risk among ATM heterozygotes.

### ACKNOWLEDGEMENTS

This project was supported by the Institutes Gustave Roussy, Villejuif and Curie, Paris, the Ligue Nationale Contre le Cancer, the Comité des Hauts-de-Seine de la Ligue Contre le Cancer, the Service de Radioprotection d'Electricité de France, INSERM. We are very indebted to the physicians who helped us contact families with AT children: C Billard, M-T Boguais, J-P Gout, B Leheup, N Philip and J-P Pollet. We are very grateful to all the participating families. We would like to thank Josyane Le Calvez for technical assistance and Diane Mathewson for linguistic revision of the manuscript.

### REFERENCES

Aitken J, Bain C, Ward M, Siskind V and MacLennan R (1995) How accurate is self-reported family history of colorectal cancer? *Am J Epidemiol* **141**: 863–871

Appleby JM, Barber JBP, Levine E, Varley JM, Taylor AMR, Stankovic T, Heighway J, Warren C and Scott D (1997) Absence of mutations in the ATM gene in breast cancer patients with severe responses to radiotherapy. *Br J Cancer* **72**: 1546–1549

Athma P, Rappaport R and Swift M (1996) Molecular genotyping shows that ataxia-telangiectasia heterozygotes are predisposed to breast cancer. *Cancer Genet Cytogenet* **92**: 130–134

Bebb G, Glickman B, Gelmon K and Gatti R (1997) 'AT risk' for breast cancer. *Lancet* **349**: 1784–1785

Benhamou E, Laplanche A, Wartelle M, Faivre J, Gignoux M, Menegoz F, Robillard J, Schaffer P, Schraub S and Flamant R (1990) Incidence des cancers en France 1978–1982. In *Statistiques de santé*, INSERM (ed). INSERM: Paris

Bishop DT and Hopper J (1997) AT-tributable risk? *Nat Genet* **15**: 226

Børresen AL, Andersen TI, Tretli S, Heiberg A and Moller P (1990) Breast cancer and other cancers in Norwegian families with ataxia-telangiectasia. *Genes Chromosomes Cancer* **2**: 339–403

Breslow NE and Day NE (1987) The design and analysis of cohort studies. In *Statistical Methods in Cancer Research*, Davis W (ed). IARC: Lyon

Coleman M, Douglas A, Hermon C and Peto J (1986) Cohort study analysis with a Fortran computer program. *Int J Epidemiol* **15**: 134–137

De Vathaire F, Koscielny S, Rezvani A, Laplanche A, Estève J and Ferlay J (1996) Estimation de l'incidence des cancers en France 1983–1987. In *Statistiques de santé*, INSERM (ed). INSERM: Paris

Easton DF (1994) Cancer risks in A-T heterozygotes. *Int J Radiat Biol* **6**: S177–182

FitzGerald MG, Bean JM, Hegde SR, Unsal H, MacDonald DJ, Harkin DP, Finkelstein DM, Isselbacher KJ and Haber DA (1997) Heterozygous ATM mutations do not contribute to early onset of breast cancer. *Nat Genet* **15**: 307–310

Kelsey JL and Horm-Ross PL (1993) Breast cancer: magnitude of the problem and descriptive epidemiology. *Epidemiol Rev* **15**: 7–16

Laake K, Odegard A, Andersen TI, Bukholm IK, Kaeresen R, Nesland JM, Ottestad L, Shiloh Y and Børresen-Dale AL (1997) Loss of heterozygosity at 11q23.1 in breast carcinomas: indication for involvement of a gene distal and close to ATM. *Genes Chromosomes Cancer* **18**: 175

Morrell D, Chase CL and Swift M (1990) Cancers in 44 families with ataxia-telangiectasia. *Cancer Genet Cytogenet* **50**: 119–123

Parkin DM, Muir CS, Whelan SL, Gao YT, Ferlay J and Powel J (1992) *Cancer Incidence in Five Continents*, Vol 5. IARC: Lyon

Pippard EC, Hall AJ, Barker DJ and Bridges BA (1988) Cancer in homozygotes and heterozygotes of ataxia-telangiectasia and xeroderma pigmentosum in Britain. *Cancer Res* **48**: 2929–2932

Savitsky K, Bar-Shira A, Gilad S, Rotman G, Ziv Y, Vanagaite L, Tagle DA, Smith S, Uziel T, Sfez S, Ashkenazi M, Pecker I, Frydman M, Harnik R, Pantanjali SR, Simmons A, Clines GA, Sartiel A, Gatti RA, Chessa L, Sanal O, Lavin MF, Jaspers NGJ, Taylor AMR, Arlett CF, Miki T, Weissman SH, Lovett M, Collins FS and Shiloh Y (1995) A single ataxia-telangiectasia gene with a product similar to PI-3 kinase. *Science* **268**: 1749–1753

Shayeghi M, Seal S, Regan J, Collins N, Barfoot R, Rahman N, Ashton A, Moohan M, Wooster R, Owen R, Bliss JM, Stratton ME and Yarnold J (1998) Heterozygosity for mutations in the ataxia telangectasia gene is not a major cause of radiotherapy complications in breast cancer patients. *Br J Cancer* **78**: 922–927

Stankovic T, Kidd AMJ, Sutcliffe A, McGuire GM, Robinson P, Weber P, Bedenham T, Bradwell AR, Easton DF, Lennox GG, Haites N, Byrd PJ and Taylor AM (1998) ATM mutations and phenotypes in ataxia-telangiectasia families in the British Isles: expression of mutant ATM and the risk of leukemia, lymphoma, and breast cancer. *Am J Hum Genet* **62**: 334–345

Swift M, Reitnauer PJ, Morrell D and Chase CL (1987) Breast and other cancers in families with ataxia-telangiectasia. *N Engl J Med* **316**: 1289–1294

Swift M, Morrell D, Massey RB and Chase CL (1991) Incidence of cancer in 161 families affected by ataxia-telangiectasia. *N Engl J Med* **325**: 1831–1836

Theis B, Boyd N, Lockwood G and Trickler D (1994) Accuracy of family history in breast cancer patients. *Eur J Cancer Prev* **3**: 321–327

*© 1999 Cancer Research Campaign*
